# PDMAEMA/Polyester Miktopolymers: Synthesis via In-Out Approach, Physicochemical Characterization and Enzymatic Degradation

**DOI:** 10.3390/ma14051277

**Published:** 2021-03-08

**Authors:** Maria Kupczak, Anna Mielańczyk, Dorota Neugebauer

**Affiliations:** Department of Physical Chemistry and Technology of Polymers, Faculty of Chemistry, Silesian University of Technology, M. Strzody 9, 44-100 Gliwice, Poland; dorota.neugebauer@polsl.pl

**Keywords:** (bio)degradable polyesters, miktopolymers, enzymatic degradation, PDMAEMA

## Abstract

Synthesis, physicochemical characterization, and the enzymatic degradation of the amphiphilic miktoarm star-shaped polymers is reported herein. First, star-shaped macroinitiators, based on *N,N*′-dimethylaminoethyl methacrylate (DMAEMA) and glycerol dimethacrylate (GDMA) ((PDMAEMA)_n_-PGDMA), were synthesized. Due to the presence of hydroxyl groups in the macroinitiator core, polyesters such as poly(ɛ-caprolactone) (P(ɛ-CL)), polylactide (PLA) and poly(lactide-*co*-glycolide) (PLGA) were synthesized using ring opening polymerization (ROP). Comprehensive degradation studies on enzymatic degradation, using a lipase from *Pseudomonas cepacia*, were performed. Enzymatic degradation was monitored by weight measurements and nuclear magnetic resonance spectroscopy (^1^H NMR). The fastest degradation rate was observed for the polymer with the lowest molecular weight. Amphiphilic miktopolymers may find application as biomaterials for long- or mid-term period drug-delivery systems.

## 1. Introduction

Biodegradable materials, due to properties such biodegradability, non-immunogenicity, and biocompatibility with living tissues, are used, among others, in the biomedical field. Aliphatic polyesters belong to the group of the most dynamically developing polymer materials, showing degradability in biological systems. These polyesters are both naturally occurring and synthetically produced [1,2,3,4].

The speed of the biodegradation process depends on many factors, such as the pH of the environment, the presence of microorganisms, and the specific properties of the polymers used [5]. Molecular weight, stereochemistry, crystallinity, hydrophobic and hydrophilic interactions, and copolymerization also have a significant impact on the speed of the biodegradation process [6,7,8].

Among the aliphatic polyesters, poly(ɛ-caprolactone) P(ɛ-CL), polylactide (PLA), polyglycolide (PGA), and a copolymer of lactide and glycolide (poly(lactide-*co*-glycolide) (PLGA)) can be distinguished due to their excellent biocompatibility, biodegradability and mechanical strength. These polymers have been approved by the United States Food and Drug Administration (US FDA) for use in tissue engineering and drug delivery [9]. P(ɛ-CL) is a semi-crystalline polyester with a melting point of 58–65 °C, a T_g_ between 65 °C and −60 °C, and a high solubility in organic solvents. [10] Due to its low degradation rate, P(ɛ-CL) is used as a material for long-term controlled drug delivery. [11] Mixtures of polycaprolactone with other polymers alter the rate of degradation, in addition to many other properties, which broadens the potential to use P(ɛ-Cl) copolymers in a range of different applications [12]. Poly(d,l-lactide) (PDLLA) is an amorphous polymer, due to the random positions of the two isomeric forms along the polymer chain, and has a T_g_ of approximately 50–60 °C [10]. The rate of degradation depends on the molecular weight and the crystallinity of the polymer as well as the porosity of the matrix. Relative to PGA, PDLLA is more hydrophobic, which translates into a slower degradation rate [13,14]. PGA is a highly-crystalline polymer with a melting point of 200–233 °C and a glass transition temperature (T_g_) of approximately 35–45 °C [10]. Due to its high crystallinity, PGA shows good mechanical properties but also very low solubility, in common organic solvents. Despite its many advantages, the use of PGA is limited due to the high rate of polymer degradation and low solubility, combined with the accumulation of acidic degradation products that can lead to inflammatory reactions. In the case of PLGA, the mechanical and degradation properties are adjusted by the appropriate monomer ratio [15,16].

Degradation of polyesters can take place biologically through a range of enzymes, such as lipase, proteinase K, pronase and bromelain, as well as chemically through both transesterification and hydrolysis in either acidic or basic solutions [17,18]. Gan et al. investigated the enzymatic degradation of P(ε-CL)/PDLLA blend films in a phosphate buffered saline (PBS) containing *Pseudomonas* (PS) lipase. The degradation was stopped after 6 days, when the film weight loss reached 50%. Studies have shown that only PCL in the blends was degraded [19]. Liu et al. investigated the enzymatic degradation of PLLA, PCL and PLLA/PCL blend films (PLLA/PCL composition 75/25, 50/50 and 25/75), using proteinase K and *Pseudomonas cepacia* lipase. They showed that PLLA amorphous domains are degraded using proteinase K; however, this enzyme did not degrade PCL and crystalline PLLA. Unlike proteinase K, *PS* lipase degraded both crystalline and amorphous PCL, but not PLLA [1]. Lin et al. investigated the enzymatic degradation of polycaprolactone-*graft*-polyethylene glycol (PCL-*g*-PEG) hydrogels in a PBS solution containing *PS* lipase over a period of 10 days. Depending on the amount of lipase, hydrogels degraded from three (1 mg/mL lipase) to 10 days (0.05 mg/mL lipase) [20]. In turn, Dong et al. degraded the terpolymer of L-lactide/1,3-trimethylene carbonate (TMC)/glycolide (P(LLA-TMC-GA)) and its composites with PLGA fibers. The degradation was performed at 37 °C in Tris buffer (pH 8.5) containing proteinase K for 30 days. They found that the rate of enzymatic degradation of PLLA-TMC-GA terpolymers with the dominant LLA component was affected by both the mean LLA block length and crystallinity [21]. Ding et al. investigated the enzymatic degradation of amphiphilic multi-block poly(ε-caprolactone urethane)s, depending on the amount of poly(ethylene glycol) (PEG) addition. The degradation was carried out in PBS solution, with the addition of AK lipase. The rate of degradation slightly increased with the content of PEG, which yielded increased hydrophilicity, reduced crystallinity and reduced melting points [22]. Jiang et al. studied enzymatic degradation of single PCL crystals and amphiphilic PCL-*b*-PEO-b-PCL and PCL-b-PEO-FG (functional groups FG = NH_2_, OCH_3_). The degradation was performed in PBS, with the addition of PS lipase [23]. Blackwell et al. investigated the enzymatic degradation of star-shaped PCL with various central units. Potassium phosphate buffer (0.2 M) and enzymes such as PS lipase and cutiase from *Thermobifida cellulosilytica* were used for the degradation. Out of all polymers, the Y-shaped miktopolymer showed the fastest rate of enzymatic degradation, using both cutinase and lipase enzymes [24]. 

This article presents the results of enzymatic degradation of amphiphilic miktoarm star-shaped polymers containing hydrophilic (PDMAEMA) and hydrophobic (P(ɛ-CL) PLA, PLGA) segments. Polymers were obtained by an “in-out” approach. To the best of our knowledge, based on the current literature, the degradation of miktopolymers with given composition with PS lipase has not yet been investigated. The main goal of the research was to establish preliminary structure−degradation relationships.

## 2. Materials and Methods

### 2.1. Materials

Anisole (Alfa Aesar, 99%, Warsaw, Poland), methanol (Chempur, p.a., Piekary Śląskie, Poland), ethyl α-bromoisobutyrate (EBiB, Aldrich 98%, Poznań, Poland) and *N*,*N*′-dimethylaminoethyl methacrylate (DMAEMA, Aldrich, 98%, Poznań, Poland) were stored over molecular sieves in a freezer under nitrogen. Glycerol dimethacrylate (GDMA, Aldrich, Poznań, Poland) was stored in a freezer under nitrogen. ε-Caprolactone (ɛ-CL, Alfa Aesar, 99%, Warsaw, Poland) and toluene were distilled prior to use and stored over molecular sieves. Glycolide (GL, Aldrich, 99%, Poznań, Poland), 3,6-dimethyl-1,4-dioxane-2,5-dione (D,L-lactide, Aldrich, 99%, Poznań, Poland) and *N*,*N*,*N*′,*N*″,*N*″-Pentamethyldiethylenetriamine (PMDETA, Aldrich, 99%, Poznań, Poland) were used as received, without purification. Copper(I) chloride (CuCl, Fluka, 98%, Steinheim, Germany) was purified by stirring in glacial acetic acid, followed by filtration and washing with ethanol and diethyl ether. Subsequently, the solid was dried under vacuum. Tin(II) bis(2-ethylhexanoate) (Sn(Oct)_2_, Alfa Aesar, 96%, Warsaw, Poland) was distilled prior to use. Tetrahydrofuran (THF, Aldrich, HPLC, Poznań, Poland), n-heptane (Chempur, 99%, Piekary Śląskie, Poland) and methylene chloride (CH_2_Cl_2_, Chempur, 99%, Piekary Śląskie, Poland) were used as received. Lipase from *Pseudomonas cepacia* (PS Lipase, ≥30 U/mg) was purchased from Sigma Aldrich (Poznań, Poland).

### 2.2. Synthesis

#### 2.2.1. Synthesis of Star-Shaped Macroinitiator (MI)

Star-shaped macroinitiators were synthesized via atom transfer radical polymerization (ATRP).

In the first step, the ATRP reaction for DMAEMA was performed. For this purpose, the monomer (DMAEMA—5 mL, 29.67 mmol) was introduced into the Schlenk-type reactor, under a constant supply of argon gas. The solvent (anisole—75% by volume of monomer) and initiator (ethyl α-bromoisobutyrate (EBiB)—49 μL, 0.33 mmol) were then added. After the ligand (PMDETA—69 μL, 0.33 mmol) was introduced, the mixture was subjected to a freezing and degassing process in liquid nitrogen. After freezing and degassing the reaction mixture three times, the catalyst (CuCl—32.64 mg, 0.33 mmol) was added and the reactor was placed in an oil bath. The process was carried out at a temperature of 45 °C. When the DMAEMA conversion was in the targeted range (80–90%), a crosslinker (GDMA) was introduced into the reaction mixture, which was first subjected to a freezing and degassing process in liquid nitrogen. The reaction was terminated 30 min after the introduction of GDMA due to the significant increase in the viscosity of the reaction mixture. The reaction system was deactivated by supplying air to it by opening the reactor. The reaction mixture was diluted with dichloromethane and passed through an alumina chromatography column to remove the catalyst before concentrating the solution by evaporation. The polymer was precipitated in n-heptane and dried to a constant weight. 

The PDMAEMA macroinitiators (MI1 and MI2) were obtained as white powders, with yields above 90%.

IR: ν = 3650–3090 cm^−1^ (ν_OH_); ν = 3050–2840 cm^−1^ (m, ν_CH_); ν = 2840–2700 cm^−1^ (m, ν_CH_, next to the amino group); ν = 1825–1640 cm^−1^ (s, ν_C=O_); δ = 1517–1300 cm^−1^ (δ_CH_); ν = 1285–890 cm^−1^ (ν_CO_ and ν_CN_).

^1^H NMR (600 MHz, CDCl_3_, δ): 4.10–4.00 (2H, s, -O-CH_2_-); 2.61–2.53 (2H, s, -CH_2_-N(CH_3_)_2_); 2.35–2.25 (6H, s, -N(CH_3_)_2_); 2.10–1.75 (2H, m, -CH_2_-); 1.15–0.80 (3H, m, -CH_3_).

#### 2.2.2. Synthesis of Miktopolymers

Due to the presence of hydroxyl groups in the core of the star macroinitiator, the ROP reaction of selected cyclic esters was carried out.

A cyclic ester (ε-CL, LA or LA and GA) and a macroinitiator ((PDMAEMA)_n_-PGDMA) were placed in the Schlenk reactor. Following this process, the solvent (toluene for LA and LA and GA—9% by weight of monomer) and the catalyst (Sn(Oct)_2_) were introduced. The reactor was placed in an oil bath at 130 °C (ɛ-CL) or 90 °C (LA and LA and GA). The reaction was complete after 1.5 h (ɛ-CL) or 22 h (LA and LA and GA). After termination of the reaction, the reaction mixture was diluted with dichloromethane. The crude product was precipitated in cold methanol and dried to a constant weight. The last step was repeated twice to remove the unreacted macroinitiator.

The resulting miktopolymers were white powders with yields above 80%.

IR: ν = 3650–3090 cm^−1^ (ν_OH_); ν = 3050–2840 cm^−1^ (m, ν_CH_); ν = 2840–270 cm^−1^ (m, ν_CH_, next to the amino group); ν = 1825–1640 cm^−1^ (s, ν_C=O_); δ = 1517–1300 cm^−1^ (δ_CH_); ν = 1285–890 cm^−1^ (ν_CO_ and ν_CN_).

^1^H NMR (600 MHz, CDCl_3_, δ) for Miktopolymer1 (MP1): 4.10–4.00 (2H, t, -O-CH_2_-); 3.65–3.60 (2H, -CH_2_-OH); 2.60–2.54 (2H,s, -CH_2_-N(CH_3_)_2_); 2.35–2.25 (2H, m, -C(O)-CH_2_-); 1.68–1.60 (4H, m, -C(O)-CH_2_-CH_2_-CH_2_-CH_2_-); 1.41–1.34 (2H, m, -C(O)-CH_2_-CH_2_-CH_2_-CH_2_-); 1.15–0.80 (3H, m, -CH_3_).

^1^H NMR (600 MHz, CDCl_3_, δ) for Miktopolymer2 (MP2): 5.28–5.10 (1H, m, -CH(CH_3_)-O-); 4.22–4.44 (1H, q, -CH(CH_3_)-OH); 1.62–1.44 (3H, m, -CH_3_).

^1^H NMR (600 MHz, CDCl_3_, δ) for Miktopolymer3 (MP3): 5.28–5.10 (1H, m, -CH(CH_3_)-O-); 4.92–4.56 (2H, m, -CH_2_-O-); 4.22–4.44 (1H, q, -CH(CH_3_)-OH); 3.76–3.79 (2H, m, -CH_2_-OH); 1.62–1.44 (3H, m, -CH_3_).

The amounts of the reagents used in the reaction are shown in Table 1.

### 2.3. Characterization

#### 2.3.1. Nuclear Magnetic Resonance (^1^H NMR)

^1^H NMR spectra of the synthesized polymers solutions in deuterated chloroform (CDCl_3_) were collected using a Varian Inova 600 MHz spectrometer (Palo Alto, CA, USA) at 25 °C, with TMS as an internal standard. The samples were prepared by dissolving about 10 mg of the test substance in 0.5 cm^3^ CDCl_3_.

#### 2.3.2. Attenuated Total Reflectance-Fourier Transform Infrared Spectroscopy (ATR-FTIR) 

ATR-FTIR analysis was carried out with a Perkin-Elmer Spectrum Two 1000 FT-IR Infrared Spectrometer with an option of attenuated total reflection (ATR) (Perkin Elmer, Waltham, MA, USA). Spectra were recorded at 16 scans per spectrum and 4 cm^−1^ resolution in the range of 4000–400 cm^−1^.

#### 2.3.3. Differential Scanning Calorimetry (DSC)

Thermal analysis was performed on the obtained compounds before and after degradation using Mettler-Toledo 821e STAR and Mettler-Toledo DSC3 differential scanning calorimeters equipped with an XS105DU analytical balance (Greifensee, Switzerland).

DSC measurements were performed before and after degradation. A total of 10 mg of the sample was used for measurements. The samples were placed in 40 μL aluminum standard crucibles with a lid and pin. The temperature range was from −5 °C to 100 °C (MI1 and MI2), −90 °C to 100 °C (MP1) and −30 °C to 200 °C (MP2, MP3), with a heating rate of 10 °C per minute.

The melting enthalpies for the MP1 were determined. Equation (1) was used to calculate the crystallinity of PCL.

X_c_(%) = (ΔH_m_/ΔH_m_°) × 100%, (1)

where ΔH_m_ is the enthalpy of melting and ΔH_m_° is the enthalpy of melting of the 100% crystalline polymer (139 J/g) [24,25].

#### 2.3.4. Gel Permeation Chromatography/Size Exclusion Chromatography (GPC/SEC)

The molecular weight distributions (Đ) and average molecular weights (M_n, SEC_) were determined by a size exclusion chromatograph (SEC, 1100 Agilent 1260 Infinity) (Santa Clara, CA, USA). The instrument had an isocratic pump, autosampler, degasser, column thermostat and MDS RI Detector differential refractometer. Data analysis software Addon Rev. was used for data collection and processing B.01.02 (Agilent Technologies). Based on a calibration using linear polystyrene standards (580–300,000 g/mol), the molecular weight was calculated. For separation, a pre-column guard 5 µm 50 × 7.5 mm and two columns (PLGel 5 µm MIXED-C 300 × 7.5 mm and PLGel 5 µm MIXED-B 300 × 7.5 mm) were used. The measurements were carried out in THF (HPLC grade) as the solvent at 40 °C with a flow rate of 0.8 mL/min.

#### 2.3.5. Enzymatic Degradation

Enzymatic degradation was carried out for obtained miktopolymers. First, approximately 10 +/−0.5 mg of each polymeric sample (white dry powder) was weighed in glass vials. In addition, to these vials 0.1 mg of PS Lipase and 5 mL of PBS solution at pH 7.4 with addition of sodium azide (23 μM), which protected the buffer against microbial growth, were added. The vials and their contents were sealed and placed on a shaker with 150 rpm at a temperature of 37 +/−2 °C. Samples were taken out of the incubator at predetermined time intervals, i.e., 1, 3, 5, 7, 14, 21 and 28 days, then frizzed and lyophilized. Samples were analyzed in triplicate. The degree of polymer degradation was calculated from the ^1^H NMR spectroscopy, and the theoretical values of the number average molecular weight of the degradable polyester chains were determined. In case of MP1 and MP2, the degree of polymerization (DP) of the ε-CL or LA was calculated based on the signal from polyester and the signal from the end group. In the case of P(ɛ-CL), signals from the methylene group in the main chain (*δ* = 4.10–4.00 ppm) and signals from the methylene group connected to the hydroxyl end group (*δ* = 3.65–3.60 ppm) were used. Whereas for PLA, the signal from methine group at shifts *δ* = 5.28–5.1 ppm (repeating unit) and *δ* = 4.28–4.30 ppm (end group) was taken into account. 

In case of MP3, the DP of LA and GA was calculated from the ^1^H NMR spectrum of the sample taken from the reaction mixture at the end of the reaction. 

## 3. Results

### 3.1. Characterization of Polymers

**Figure 1:**
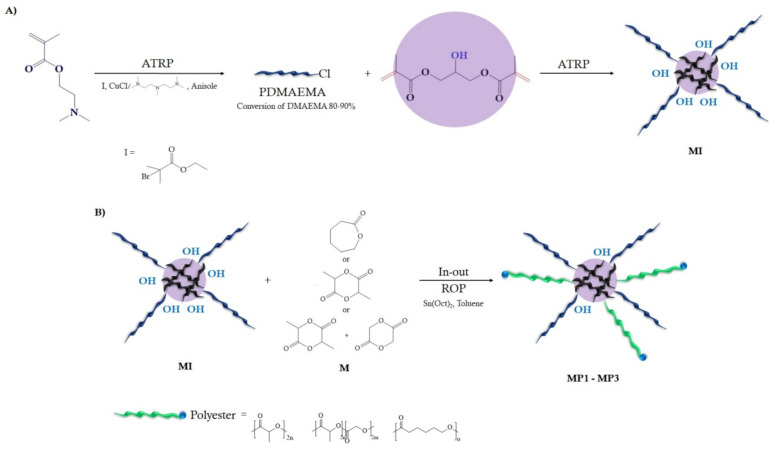
The synthesis of macroinitiators (**A**) and miktopolymers (**B**).

Two star-shaped macroinitiators of [(PDMAEMA)_n_-PGDMA] were synthesized by ATRP with a standard initiator EBiB in the presence of the catalyst complex CuCl/PMDETA in anisole at 45 °C. The presence of hydroxyl groups in their core allowed the use of a star-shaped macroinitiator in the synthesis of polyesters such as P(ɛ-CL), PLA and a PLGA copolymer using ROP. Thanks to this, the obtained amphiphilic miktoarm star-shaped polymers have in their structure biodegradable polyester blocks as well as nonbiodegradable polymethacrylate blocks. This procedure is schematically presented in Figure 1.

**Figure 2:**
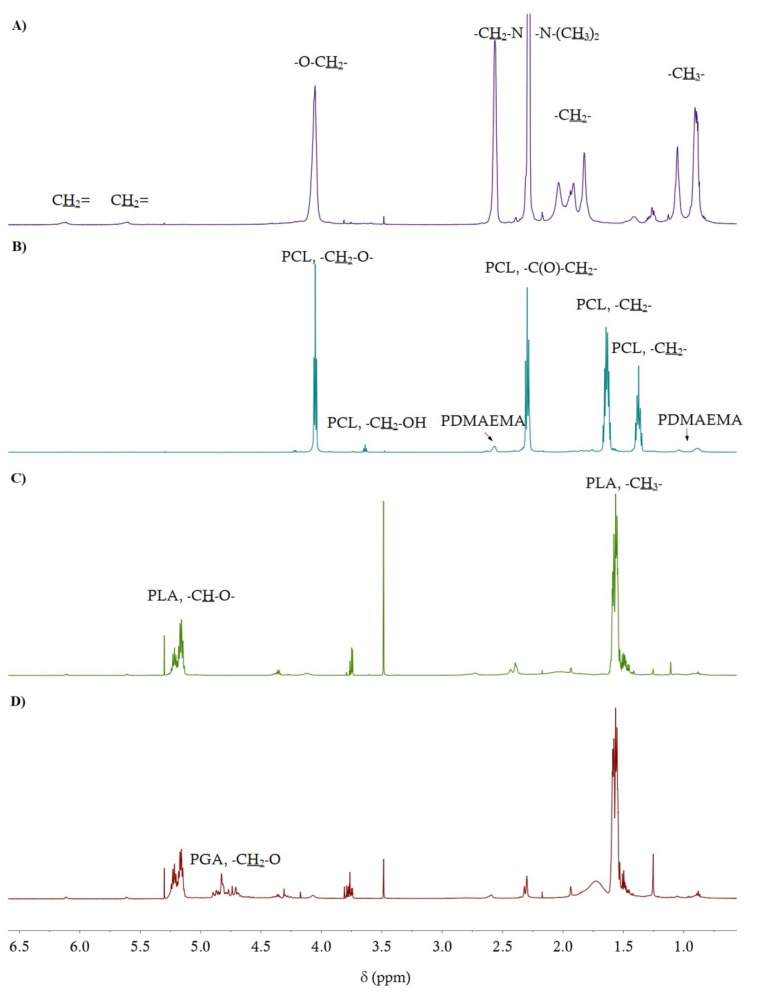
^1^H NMR (600 MHz, CDCl_3_) spectra of MI (**A**) and MP1 (**B**), MP2 (**C**) and MP3 (**D**) miktopolymers before degradation.

The characterization of the obtained polymers is presented in Table 2. The structures of the obtained macroinitiators and miktopolymers were confirmed by spectroscopic methods, ^1^H NMR and ATR-FTIR. Figure 2 shows signals from groups of protons present in the polymers. The signals from poly(di)methacrylates were less intense than the signals from polyesters. This indicates a greater proportion of the hydrophobic (polyester) fraction in the miktopolymer molecule, compared to the hydrophilic (polymethacrylate) fraction. The hydrophobic fraction (F_hydrophobic_) for each miktopolymer was calculated from the NMR spectra, where the intensities of the characteristic signals from individual polyesters were compared to the signal intensity from PDMAEMA. Based on the calculated degree of polymerization (DP) of cyclic (di)esters in P(ɛ-CL), PLA and PLGA, before and after degradation, the theoretical number-average molecular weight (M_n, NMR_) of the polyester part was determined.

**Figure 3:**
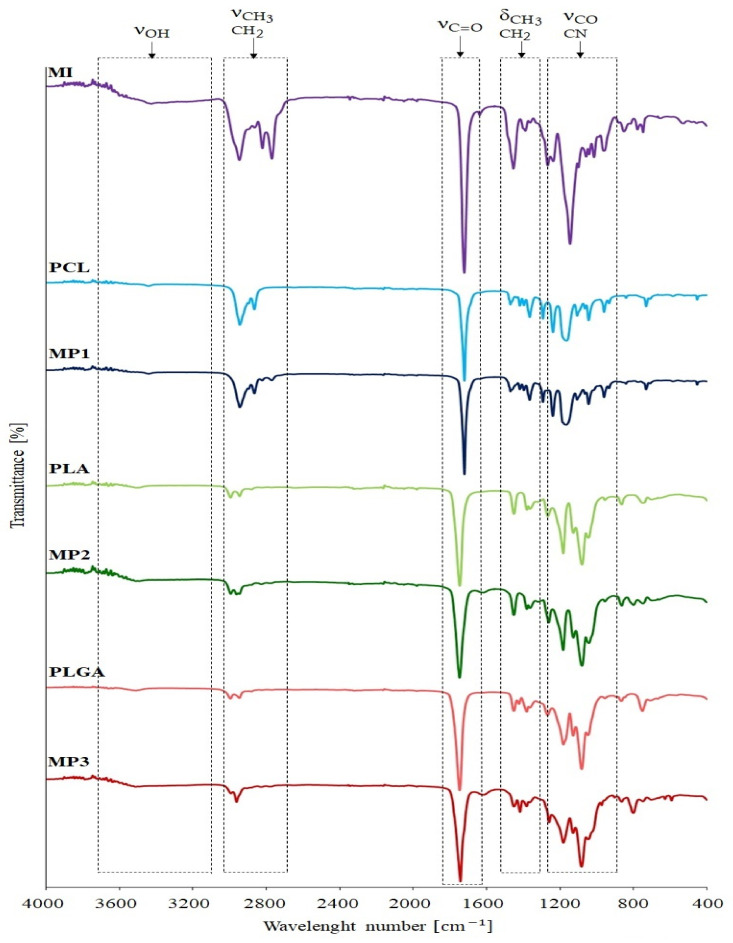
ATR-FTIR spectra of obtained miktopolymers before degradation and pure polyesters.

On the ATR-FTIR spectra of the obtained miktopolymers, a gradual decrease in the intensity of the vibration bands belonging to PDMAEMA was observed due to the high proportion of polyester fraction in the obtained miktopolymers (Figure 3). Moreover, the broadening of the band of carbonyl groups (C=O) in the range between 1760 cm^−1^ and 1750 cm^−1^ for miktopolymers, in comparison to the macroinitiator and corresponding polyester, was noticed. This results were in good agreement with ^1^H NMR spectra.

**Figure 4:**
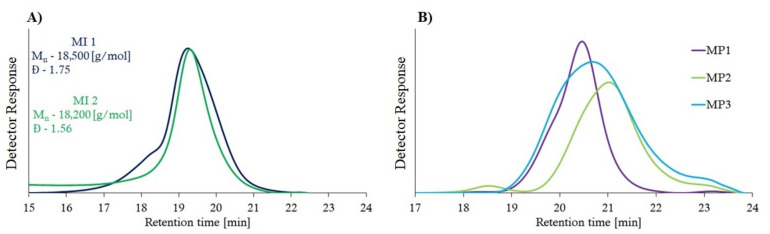
SEC traces of macroinitiators (**A**) and miktopolymers (**B**) before degradation.

Molecular weights were determined by SEC analysis. The obtained miktopolymers had lower values of dispersion (Đ) and number average molecular weights (M_n_) compared to their macroinitiators (Table 2, Figure 4). 

**Figure 5:**
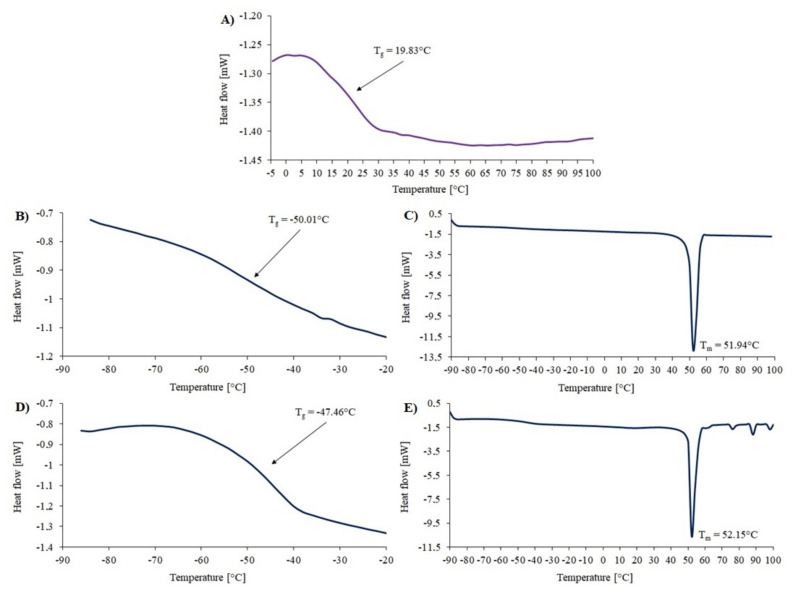
DSC thermogram for MI 1 (**A**) and MP1 before (**B**,**C**) and after (**D**,**E**) degradation.

Thermal analysis was also performed for the obtained compounds before and after degradation using DSC. For MP1 before degradation, the glass transition temperature (T_g_) and melting point (T_m_) was determined. The value of the T_g_ (−50.01 °C) narrowly deviated from the temperature in the literature for the P(ɛ-CL) arms, which varies from −65 °C to −60 °C. The value of the T_m_ slightly deviated from the literature data for P(ɛ-CL) homopolymer (T_m_ = 58–65 °C), probably because when morphological confinement increases, the crystallization and melting temperature values may decrease [26,27] (Figure 5) [10]. After the enzymatic degradation, the degree of crystallinity of P(ε-CL) decreased from X_c_ = 50% to X_c_ = 37%. Additionally, the T_m_ and T_g_ increased. 

**Figure 6:**
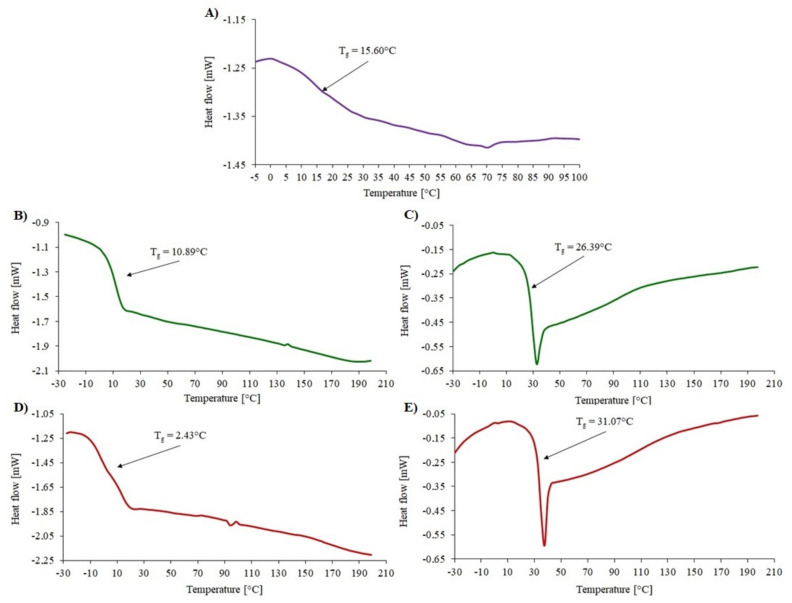
DSC thermogram for MI 2 (**A**), MP2 before (**B**) and after (**C**) degradation and MP3 before (**D**) and after (**E**) degradation.

Before degradation, a miktopolymer containing a PLGA copolymer in its structure had a lower T_g_ (2.43 °C) than a polymer with PLA (10.89 °C) (Figure 6). The lower glass transition temperature may be due to the presence of GA units in the sequence structure [27]. According to the literature, the T_g_ of PGA is 35–45 °C. The theoretical T_g_ for the PLGA copolymer is 50 °C, while the resulting T_g_ obtained by DSC is 2.43 °C. This may indicate that a branched miktopolymer was obtained. Therefore, after degradation, the T_g_ value for MP2 and MP3 increased. The T_g_ depends on chain flexibility, molecular weight, and cross-linking. In that case, it seems that the T_g_ increases after degradation due to the reduction of the polyester fraction caused by its degradation [28]. Moreover, the relaxation enthalpy appeared at the thermograms of MP2 and MP3 after degradation.

### 3.2. Enzymatic Degradation

**Figure 7:**
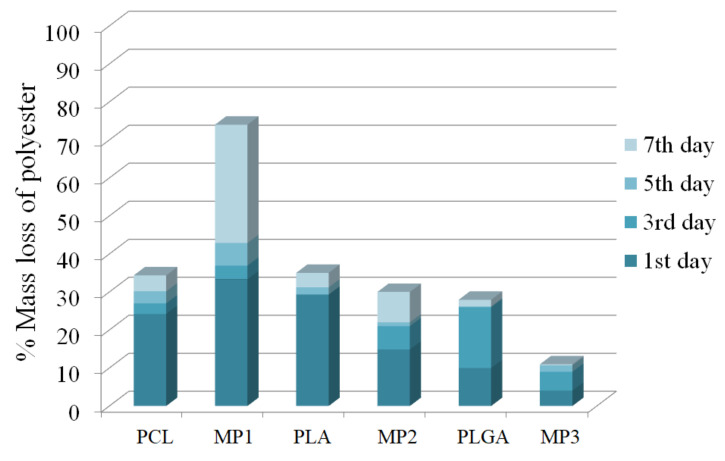
Percentage of mass loss of polyesters as well as a polyester segment in miktostars during the course of enzymatic degradation.

In our research, enzymatic degradation with PS lipase was performed for obtained miktopolymers in 0.01 M PBS at a pH 7.4 and a temperature of 37 °C. The number-average molecular weight of the polyester segment within a given miktopolymer was calculated from the ^1^H NMR spectra and presented as the percentage of the mass loss. Moreover, in order to elucidate the impact of PDMAEMA segment on the degradation process, the linear polyesters were also examined as positive controls. At this point, it is worth noting that, due to the lack of exchange of the enzyme solution, the degradation process was naturally suppressed by the decrease in enzyme activity. Figure 7 shows that the MP1 with P(ɛ-CL) arms degraded to the greatest extent, and its weight loss after 7 days of degradation was approximately 70%. In comparison, the mass loss of the miktopolymer with PLA arms was approximately 30%; with PLGA arms, it showed the lowest mass loss, approximately 10%. Taking into account the first 24 h of degradation, the presence of PDMAEMA segment increased the degradation rate for P(ɛ-CL) arms. Comparing the results obtained for MP2 and MP3 to the corresponding linear polyester, the extent of the % mass loss of polyester was lower for miktopolymers.

## 4. Discussion

The amphiphilic miktoarm star-shaped polymers, based on poly(di)methacrylates and polyesters, were synthesized and characterized. The analysis of ^1^H NMR and ATR-FTIR indicates that hydrophobic chains dominate the polymer compositions, which, in turn, affects the solubility of the obtained polymers. The molecular weight distribution of the polymers was in the range of 1.2–1.5, which proves that they were obtained in a *δ* controlled manner. The highest molecular weight distribution value is found in a polymer that has polyglycolide in its structure. The difference of hydrodynamic volumes between PS standards and branched DMAEMA/polyester copolymers caused the decrease of M_n, SEC_ values, which are apparent molecular weights. Moreover, there were two different macroinitiators used in the synthesis of miktopolymers. The MI1 has shorter PDMAEMA arms than MI2; thus the M_n, SEC_ is slightly higher. The obtained miktopolymers had lower M_n,SEC_ values compared to their macroinitiators (Table 2, Figure 4) due to the higher affinity of hydrophobic polyester part to the stationary phase of the column (PS crosslinked with divinylbenzene). The thermal analysis showed that the presence of compounds with different physicochemical properties in the polymer structure is of great importance in the determination of their thermal properties, such as the T_g_ and T_m_.

Enzymatic degradation is affected by the way the enzyme interacts with the polymer chains. Physicochemical properties such as molecular weight, structure, crystallinity and properties of the enzyme, i.e., activity, concentration and stability, also influence the rate of degradation [29]. The thermograms of miktopolymers after degradation showed that the T_m_ slightly increased. Enzymatic degradation primarily occurring in amorphous regions of the polyester chains should cause a decrease of T_g_. In our case, the T_g_ increased, and for MP2 and MP3 samples, the additional peak from relaxation enthalpy appeared. This might be a result of the increase of the PDMAEMA fraction and slight reordering of the amorphous phase of the sample [30]. The degree of crystallinity decreased to 37% after seven days of degradation. The decrease in the degree of crystallinity could be caused by several factors, including a high initial degree of crystallinity, the appearance of oligomers or a decrease in the length of the crystallizable block [31,32]. However, there are articles in which researchers claimed that the crystallinity of polyesters increases with increasing degradation time [33,34]. Unfortunately, the phase separation in thermograms of miktopolymers was not observed due to the overlap of T_g_ signal from PDMAEMA part with the T_m_ signal from polyester arms.

The enzymatic degradation in measurement of both the average weight loss of the sample and % mass loss based on ^1^H NMR spectroscopy showed that P(ɛ-CL) degrades faster than PLA and PLGA. Blackwell et al. investigated enzymatic degradation using PS lipase of star P(ɛ-CL) with different central units. They showed that the polymer degrades faster with increasing content of the hydrophilic compound [24]. Moreover, Liu et al. proved that PS lipase can degrade both amorphous and crystalline PCL but cannot degrade PLLA [1]. Since we used 50:50 racemic mixture of D- and L-lactide for the synthesis of MP2 and MP3, the obtained miktopolymers underwent degradation to a lesser extent. In the case of PLA and PLGA, the degradation rate largely depends on the copolymer composition. Since, according to the literature, the presence of GA repeating units hinders the degradation of PLGA by PS lipase, the degradation rate of MP2 and MP3 was lower in comparison to MP1, due to the presence of D-lactide or glycolide [29,35]. In the case of our research, the presence of the hydrophilic segment in the polymer molecule affected the degradation rate. Moreover, degradation of polyesters can take place by autocatalytic hydrolysis [36]. We assume that hydroxyacids, which are the products of polyester degradation, enhanced protonation of the secondary amine groups in PDMAEMA, causing the electrostatic repulsion and higher affinity of PDMAEMA arms to water molecules. The increase in mass loss of PCL in MP1 and PLA in MP2 after seven days of the experiment can be explained by loosening of the miktopolymers structure and improved contact with the enzyme (Figure 7).

Degradable polymers degrade at a range of rates, due to different degrees of crystallinity, different molecular weights, the presence of cross-linking bonds, and hydrophobic or hydrophilic character. Additionally, the presence of arms with amine functional groups in the miktopolymers structure may facilitate the process of polyester degradation and increase the spectrum of potential applications of the obtained polymers.

## 5. Conclusions

The in-out approach allowed us to obtain amphiphilic semi-degradable miktoarm star-shaped polymers. Star-shaped macroinitiators were successfully synthesized by the arm-first method (ATRP), which, owing to the presence of hydroxyl groups in the core, allowed for the ROP of selected (di)cyclic esters to be carried out with high efficiency. Due to the presence of a biodegradable segment, the enzymatic degradation of miktopolymers in the presence of PS lipase was carried out. The analysis of the products obtained after enzymatic degradation of miktopolymers showed that the fastest degradation rate was observed for the miktopolymer with PCL arms. This resulted from the high content of the hydrophilic fraction in the miktopolymer in comparison to the rest of the polymers as well as the selectivity of the PS lipase toward degradation of PCL and PLLA. The additional factor influencing the degradation rate was the content of GA units in PLGA miktpolymer. The results indicate that the capability of the polymeric material for degradation in specific conditions can be modified by proper designing of the polymeric structure and composition.

## Figures and Tables

**Table 1:** Amounts of used reagents.

Miktopolymer	ɛ-CL		LA		GA		MI		Sn(Oct)_2_	
	mL	mmol	g	mmol	g	mmol	g	mmol	μL	mmol
(MP1)	1.5	13.54	-	-	-	-	0.7	0.05	18	0.05
(MP2)	-	-	1.5	10.41	-	-	0.4	0.03	10	0.03
(MP3)	-	-	1.5	10.41	0.4	3.47	0.5	0.04	13	0.04

**Table 2:** Characterization of obtained polymers.

Miktopolymer	MI	DP_DMAEMA per arm_	DP_CL per arm_	DP_LA per arm_	DP_GA per arm_	Hydrophobic Fraction (%)	M_n, NMR_ (g/mol)	M_n, SEC_ (g/mol)	Ð
MP1	MI 1	72	28	-	-	80	40,400	7300	1.24
MP2	MI 2	78	-	27	-	94	105,700	4100	1.28
MP3 *	MI 2	78	-	15	10	95	130,200	5100	1.51

Where * LA:GA = 75:25.

## Data Availability

The data presented in this study are available on request from the corresponding author.

## References

[1] Liu L., Li S., Garreau H., Vert M. (2000). Selective enzymatic degradations of poly(L-lactide) and poly(ε-caprolactone) blend films. Biomacromolecules.

[2] Luckachan G.E., Pillai C.K.S. (2011). Biodegradable Polymers- A Review on Recent Trends and Emerging Perspectives. J. Polym. Environ..

[3] Athanasiou K.A., Niederauer G.G., Agrawal C.M. (1996). Sterilization, toxicity, biocompatibility and clinical applications of polylactic acid/polyglycolic acid copolymers. Biomaterials.

[4] Gunatillake P.A., Adhikari R., Gadegaard N. (2003). Biodegradable synthetic polymers for tissue engineering. Eur. Cells Mater..

[5] Chłopek J., Morawska-Chochół A., Szaraniec B. (2010). The influence of the environment on the degradation of polylactides and their composites. J. Achiev. Mater. Manuf.Eng..

[6] Geralt S. (2002). Degradable Polymers Principles and Applications.

[7] Sivalingam G., Chattopadhyay S., Madras G. (2003). Solvent effects on the lipase catalyzed biodegradation of poly (ε-caprolactone) in solution. Polym. Degrad. Stab..

[8] Li S., McCarthy S. (1999). Influence of crystallinity and stereochemistry on the enzymatic degradation of poly(lactide)s. Macromolecules.

[9] Jin Q., Maji S., Agarwal S. (2012). Novel amphiphilic, biodegradable, biocompatible, cross-linkable copolymers: Synthesis, characterization and drug delivery applications. Polym. Chem..

[10] Van de Velde K., Kiekens P. (2002). Biopolymers: Overview of several properties and consequences on their applications. Polym. Test..

[11] Darney P.D., Monroe S.E., Klaisle C.M., Alvarado A. (1989). Clinical evaluation of the Capronor contraceptive implant: Preliminary report. Am. J. Obstet. Gynecol..

[12] Middleton J.C., Tipton A.J. (2000). Synthetic biodegradable polymers as orthopedic devices. Biomaterials.

[13] Gupta B., Revagade N., Hilborn J. (2007). Poly(lactic acid) fiber: An overview. Prog. Polym. Sci..

[14] Lasprilla A.J.R., Martinez G.A.R., Lunelli B.H., Jardini A.L., Filho R.M. (2012). Poly-lactic acid synthesis for application in biomedical devices—A review. Biotechnol. Adv..

[15] Gunatillake P., Mayadunne R., Adhikari R. (2006). Recent Developments in Biodegradable Synthetic Polymers. Biotechnol. Annu. Rev..

[16] Ueda H., Tabata Y. (2003). Polyhydroxyalkanonate derivatives in current clinical applications and trials. Adv. Drug Deliv. Rev..

[17] Tokiwa Y., Calabia B.P., Ugwu C.U., Aiba S. (2009). Biodegradability of Plastics. Int. J. Mol. Sci..

[18] Hwan J., Ree M., Kim H. (2006). Acid- and base-catalyzed hydrolyses of aliphatic polycarbonates and polyesters. Catal. Today.

[19] Gan Z., Yu D., Zhong Z., Liang Q., Jing X. (1999). Enzymatic degradation of poly(ε-caprolactone)/poly(dl-lactide) blends in phosphate buffer solution. Polymer.

[20] Lin G., Cosimbescu L., Karin N.J., Gutwska A., Tarasevich B.J. (2013). Injectable and thermogelling hydrogels of PCL-g-PEG: Mechanisms, rheological and enzymatic degradation properties. J. Mater. Chem. B.

[21] Dong J., Liao L., Ma Y., Shi L., Wang G., Fan Z., Li S., Lu Z. (2014). Enzyme-catalyzed degradation behavior of L -lactide/trimethylene carbonate/glycolide terpolymers and their composites with poly(l-lactide-co-glycolide) fibers. Polym. Degrad. Stab..

[22] Ding M., Qian Z., Wang J., Li J., Tan H., Gu Q., Fu Q. (2011). Effect of PEG content on the properties of biodegradable amphiphilic. Polym. Chem..

[23] Jiang N., Jiang S., Hou Y., Yan S., Zhang G., Gan Z. (2010). In fl uence of chemical structure on enzymatic degradation of single crystals of PCL-b-PEO amphiphilic block copolymer. Polymer.

[24] Blackwell C., Haernvall K., Guebitz G., Groombridge M., Gonzales D., Khosravi E. (2018). Enzymatic Degradation of Star Poly(ε-Caprolactone) with Different Central Units. Polymers.

[25] Rosario F., Corradini E., Casarin S.A., Agnelli J.A.M. (2013). Effect of Gamma Radiation on the Properties of Poly(3- Hydroxybutyrate-co-3-Hydroxyvalerate)/Poly (e-Caprolactone) Blends. J. Polym. Environ..

[26] Müller A.J., María B., Arnal L. (2005). Nucleation and Crystallization in Diblock and Triblock Copolymers. Adv. Polym. Sci..

[27] Lorenzo A.T., Muller A.J., Lin M.-C., Chen H.-L., Jeng U.-S., Priftis D., Pitsikalis M., Hadjichristidis N. (2009). Influence of Macromolecular Architecture on the Crystallization of (PCL2)-b-(PS2)4-Miktoarm Star Block Copolymers in Comparison to Linear PCL-b-PS Diblock Copolymer Analogues. Macromolecules.

[28] Menczel J.D., Prime R.B., Menczel J.D., Prime R.B. (2009). Polymers Thermal Analysis of Fundamentals and Applications.

[29] Kemme M., Prokesch I., Heinzel-Wieland R. (2011). Comparative study on the enzymatic degradation of poly(lactic-co-glycolic acid) by hydrolytic enzymes based on the colorimetric quantification of glycolic acid. Polym. Test..

[30] Drogoń A., Pyda M. (2019). Badanie procesu fizycznego starzenia amorficznego polilaktydu metodą różnicowej kalorymetrii skaningowej. Polim. Polym..

[31] Fortunati E., Gigli M., Luzi F., Dominici F., Lotti N., Gzzano M., Cano A., Chiralt A., Munari A., Kenny J.M. (2017). Processing and characterization of nanocomposite based on poly(butylene/triethylenesuccinate) copolymers and cellulose nanocrystals. Carbohydr. Polymer.

[32] Gigli M., Negroni A., Soccio M., Zanaroli G., Lotti N., Fava F., Munrari A. (2013). Enzymatic hydrolysis studies on novel eco-friendly aliphatic thiocopolyesters. Polym. Degrad. Stab..

[33] Eldsater C., Erlandsson B., Renstad R., Albertsson A.-C., Karlsson S. (2000). The biodegradation of amorphous and crystalline regions in film-blown poly(ɛ-caprolactone). Polymer.

[34] Jenkins M.J., Harrison K.L. (2008). The effect of crystalline morphology on the degradation of polycaprolactone in a solution of phosphate buffer and lipase. Polym. Adv. Technol..

[35] Tokiwa Y., Calabia B.P. (2006). Biodegradability and biodegradation of poly(lactide). Appl. Microbiol. Biotechnol..

[36] Irfan S.A., Razali R., KuShaari K., Mansor N. (2017). Reaction-multi diffusion model for nutrient release and autocatalytic degradation of pla-coated controlled-release fertilizer. Polymers.

